# Role and mechanisms of mast cells in brain disorders

**DOI:** 10.3389/fimmu.2024.1445867

**Published:** 2024-08-26

**Authors:** Xuanyu Huang, Ziwei Lan, Zhiping Hu

**Affiliations:** Department of Neurology, The Second Xiangya Hospital of Central South University, Changsha, Hunan, China

**Keywords:** mast cell, brain disorders, neuroinflammation, blood-brain barrier, neuroprotective effect

## Abstract

Mast cells serve as crucial effector cells within the innate immune system and are predominantly localized in the skin, airways, gastrointestinal tract, urinary and reproductive tracts, as well as in the brain. Under physiological conditions, brain-resident mast cells secrete a diverse array of neuro-regulatory mediators to actively participate in neuroprotection. Meanwhile, as the primary source of molecules causing brain inflammation, mast cells also function as the “first responders” in brain injury. They interact with neuroglial cells and neurons to facilitate the release of numerous inflammatory mediators, proteases, and reactive oxygen species. This process initiates and amplifies immune-inflammatory responses in the brain, thereby contributing to the regulation of neuroinflammation and blood-brain barrier permeability. This article provides a comprehensive overview of the potential mechanisms through which mast cells in the brain may modulate neuroprotection and their pathological implications in various neurological disorders. It is our contention that the inhibition of mast cell activation in brain disorders could represent a novel avenue for therapeutic breakthroughs.

## 1 Introduction

Mast cells (MCs) are derived from hematopoietic progenitor cells and undergo maturation in vascular tissues, playing a role in both innate and adaptive immune responses (1). As sentinel cells of the immune system, MCs are predominantly distributed in anatomical regions that come into contact with the external environment, such as the skin, respiratory tract, gastrointestinal tract, and urinary tract (2). MCs possess the capability to degranulate and engage in cross-talk with diverse immune cells, thereby playing a pivotal role in safeguarding against pathogenic microorganisms and potential environmental threats (2, 3).

MCs are predominantly located within the vascular lumen of the brain membrane, entorhinal cortex, choroid plexus, olfactory bulb, midbrain, thalamus, and hypothalamus regions (4), where they engage in interactions with neurons, glial cells, and endothelial cells (5, 6). Furthermore, MCs are also found on the basal side of the blood-brain barrier (BBB) (6). MCs not only facilitate normal brain development and function, but also play a crucial role in the regulation of cognition and emotion (5, 7, 8). Additionally, MCs are considered the “first responders” of the brain, promptly detecting external stimuli and releasing inflammatory mediators and chemoattractants to recruit inflammatory cells in response to injury. They are pivotal in initiating, amplifying, and sustaining immune and neural responses (6). As one of the most prominent immune cell populations in the brain, MCs have emerged as a focal point of research in the context of brain disorders (9).

## 2 Mast cells and neuroinflammation

Neuroinflammation plays a crucial role in the pathogenesis and progression of various brain disorders, making it a prime target for therapeutic intervention (10, 11). Mounting evidence suggests that MCs, alongside traditional immune and inflammatory cells such as microglia and astrocytes, also contribute to the immunological and inflammatory processes within the brain (12, 13). MCs have the capacity to release cellular secretions containing immune and inflammatory mediators, such as histamine, β-tryptase, tumor necrosis factor-α (TNF-α), and interleukin-1β (IL-1β), among others (14). It is noteworthy that MCs serve as a predominant source of histamine within the brain. Indeed, more than half of the total histamine content found in the central nervous system is attributed to the degranulation of MCs (12, 15). Additionally, MCs are the sole cells in the brain that preform and store TNF-α in advance (16). MCs also express receptors and ligands for various inflammation pathways, including the protease-activated receptor-2 (PAR2)-mitogen activated protein kinase (MAPK)-nuclear factor κb (NF-κB), histamine receptors 1 (H1R)/H4R-MAPK, and phosphatidylinositol 3-kinase (PI3K)/protein kinase B (AKT)-NF-κB signaling cascades (17, 18).

**Figure 1:**
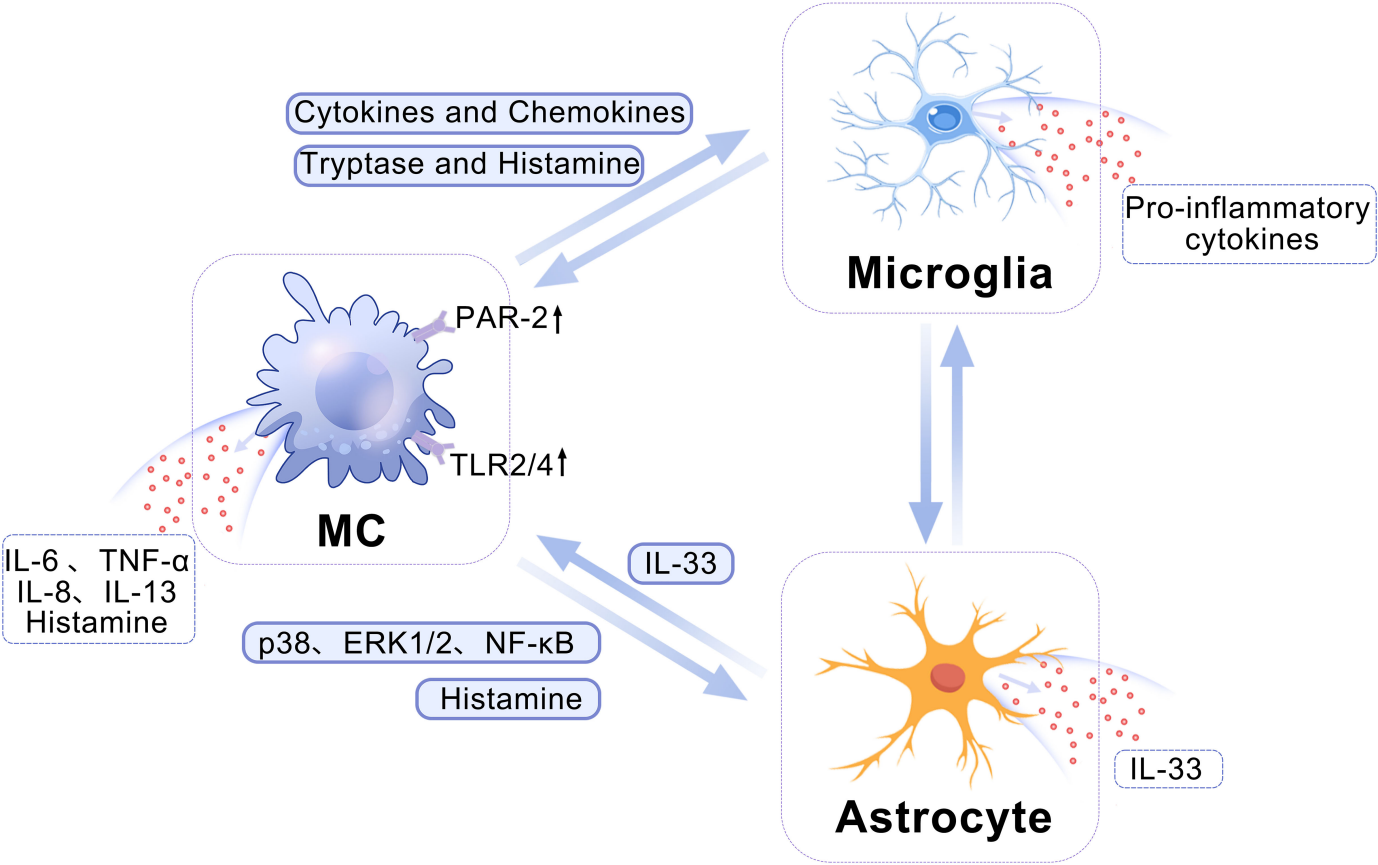
A schematic depicting the crosstalk among MCs, microglia, and astrocytes. MCs can modulate the pro-inflammatory phenotype of microglia by enhancing the expression of cytokines and chemokines. Additionally, MC-released tryptase and histamine can activate PAR-2 receptors on microglia, ultimately leading to the induction of a robust release of pro-inflammatory factors by microglia. The histamine released by MCs can stimulate the histamine receptors on astrocytes, thereby modulating their activation. Concurrently, MCs secrete diverse proteases that facilitate the release of IL-33 through activation of the p38, ERK1/2 MAPKs, and NF-κB signaling pathways in astrocytes. ERK, Extracellular-signal-regulated kinase; IL, interleukin; MAPK, mitogen-activated protein kinase; MC, mast cell; NF-κB, nuclear factor κB; PAR-2, protease-activated receptor 2.

In addition to their intrinsic functions, MCs have the potential for bidirectional signaling with microglia and astrocytes, leading to the induction and exacerbation of neuroinflammation (12) (**Figure 1**). Activated MCs can upregulate levels of cytokines and chemokines, thereby promoting the polarization of microglia towards pro-inflammatory phenotypes (19). Furthermore, MCs release proteolytic enzymes, which can activate PAR-2 receptors on microglia, triggering the release of pro-inflammatory cytokines such as TNF-α, IL-1β, and IL-6 (17, 20, 21). Notably, MCs residing in the white matter have the capacity to instigate alterations in both the morphology and function of astrocytes. This is achieved through the action of multiple proteases that originate from mast cells. These proteases can trigger the release of IL-33 from astrocytes by engaging and activating specific intracellular signaling cascades. They activate the p38, ERK1/2 MAPKs, and NF-κB signaling pathways, thereby facilitating the astrocytic response to inflammation (22). Moreover, MC-derived histamine can modulate the activity of microglia and astrocytes by binding to multiple histamine receptors on these cells (5, 17, 23, 24). In turn, activated microglia and astrocytes can reciprocally influence MCs, leading to upregulation of PAR-2 and Toll-like receptors 2 (TLR2)/TLR4 expression in MCs and triggering the release of histamine, IL-6, and TNF-α (20, 25). IL-33 generated and secreted by activated astrocytes can also activate the suppression of tumorigenicity-2 (ST-2) receptor to stimulate microglia and MCs, thereby promoting the proliferation of the former and inducing the latter to produce IL-6, IL-8, and IL-13 (26–28). In conclusion, reciprocal activation of MCs, microglia, and astrocytes through diverse signaling pathways potentiates the inflammatory response, thereby exacerbating disease prognosis.

## 3 Mast cells and neurons

**Figure 2:**
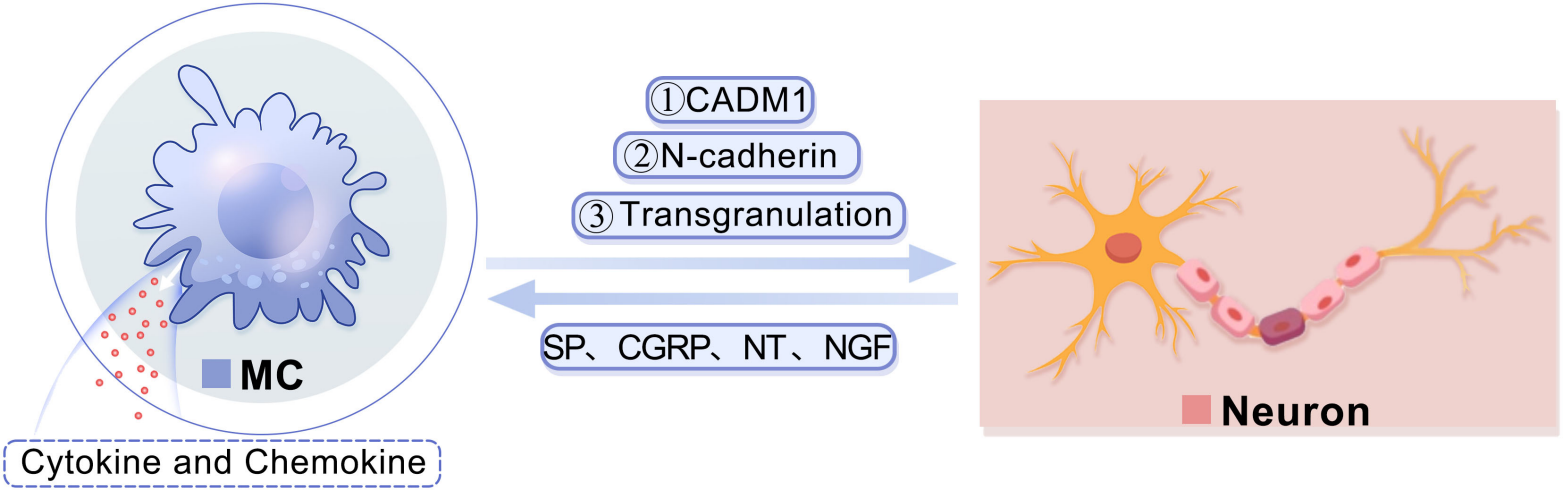
A diagram showing the interaction between MCs and neurons.MCs are capable of engaging in communication with neurons via CADM1. Furthermore, MCs have the ability to modulate the structural alterations of neuronal dendrites and synapses, facilitate the establishment and preservation of synaptic connections, and regulate synaptic adhesion by controlling N-cadherin. Additionally, MCs can release heparin into neurons through transgranulation to disrupt calcium homeostasis. Consequently, the release of various neuropeptides by neurons, such as SP, CGRP, NT, and NGF, can stimulate mast cells and prompt them to secrete cytokines and chemokines. CADM1, cell adhesion molecule-1; CGRP, calcitonin gene-related peptide; MC, mast cell; NGF, nerve growth factor; NT, neurotensin; SP, substance P.

MCs can engage with neurons via cell adhesion molecule-1 (CADM1), N-cadherin, and transgranulation (**Figure 2**). CADM1 plays a pivotal role in mediating the adhesion between MCs and neurons, facilitating information exchange between them, and is closely associated with neural immunity (29). The interaction between MCs and neurons is facilitated through synaptic-like structures, wherein N-cadherin assumes a crucial function (30). Research has demonstrated that N-cadherin participates in regulating pre- and post-synaptic structural modifications, promoting the establishment and maintenance of synaptic connections, as well as governing synaptic adhesion (30). Additionally, transgranulation enables MCs to release heparin into neurons, thereby disrupting calcium homeostasis and inhibiting neuronal responses (31). In turn, the activation of MCs can be induced by various neuropeptides such as substance P (SP), calcitonin gene-related peptide (CGRP), neurotensin (NT), and nerve growth factor (NGF) released by neurons, leading to the release of cytokines and chemokines like monocytic chemotactic protein 1 (MCP-1), IL-8, and C-C chemokine ligand 5 (CCL5) (32, 33).

Under normal physiological conditions, MCs contribute to neural protection and repair through the secretion of neuro-regulatory mediators (**Table 1**). Research has demonstrated that MCs secrete histamine, which can mitigate neuronal excitotoxicity induced by N-methyl-D-aspartatic acid (NMDA) and glutamate through the cyclic adenosine monophosphate (cAMP)/protein kinase A (PKA) pathway or by upregulating glutamate transporter currents and glutamine synthetase expression (34). Additionally, MCs are capable of promoting neuron growth by secreting NGF and inducing neuropeptide expression, lowering the firing threshold, and enhancing central nervous system transmission (35). Moreover, NGF binds to a specific receptor on the surface of microglia, enhancing membrane dynamics and endocytosis, while mediating the tyrosine kinase A pathway. This leads to an augmented phagocytic capacity for substances such as β-amyloid and others, thereby exerting neuroprotective effects (36). Under the stimulation of FcϵRI-mediated signaling, TLR ligands, and NGF, MCs synthesize and release angiogenin (37), which supports motor neuron survival and neurogenesis (38). Additionally, MCs in the hippocampus secrete serotonin, which plays a crucial role in regulating hippocampal-dependent behavior and enhancing neurogenesis (39).

**Table 1:** The principal neuroprotective mediators released by mast cells.

Mediators	Key features	Function
Histamine	More than 50% of histamine in the brain is derived from mast cells	• Suppress neuroinflammatory and inhibit glial scar formation • Mitigate NMDA and glutamate-induced neuroexcitability toxicity • Attenuate nerve injury and infarct volume following cerebral ischemia
NGF	Released during degranulation	• Facilitate neuronal growth, induce neuropeptide expression, decrease the firing threshold, and augment central nervous system transmission • Enhance microglial phagocytic function.
Angiogenin	Released by the IgE receptor crosslinking, TLR ligands, and NGF.	• Facilitate motor neuron survival and promote neurogenesis
Serotonin	Unlike humans, it is found in high concentrations in rodent mast cells	• Regulate hippocampal-dependent behavior and enhancing neurogenesis.

IgE, immunoglobulin E; NGF, nerve growth factor; NMDA, n-methyl-d-aspartic acid; TLR, toll-like receptors.

## 4 Mast cells and blood-brain barrier

The BBB serves as a highly selective and semipermeable interface between the brain parenchyma and the circulatory system, playing a pivotal role in upholding the normal function of the brain and the homeostasis of the internal environment (40). Research has revealed that MCs are frequently situated in close proximity to the active sites of matrix metalloproteinases (MMPs) (41). They influence the permeability of the BBB by relaeasing MMP. Initially, activated MMPs degrade a majority of the protein constituents within the extracellular matrix (ECM), such as collagen, elastin, fibronectin, and vitronectin (42, 43); subsequently, MMPs target cleavage sites of tight junctions (TJs) proteins, enabling brain microvascular endothelial cells (BMECs) to detach from the ECM (44); ultimately, key components of MMPs, namely MMP-2 and MMP-9, can directly break down microvascular basement membrane components, particularly type IV collagen (45, 46). Moreover, MCs secrete tryptase that are capable of activating PAR-2 receptors to enhance the expression of vascular cell adhesion molecule-1 (VCAM-1), TLR4, and TNF-α, while simultaneously reducing the expression of Occludin and Claudin-5, thereby inducing an increase in BBB permeability (47). Additionally, tryptase can directly compromise the integrity of the BBB by breaking down zonula occludens-1 (ZO-1), ZO-2, Occludin, and Claudin-5 proteins associated with TJs (48). Furthermore, MCs release substantial quantities of histamine which can bind to H1 and H2 receptors on endothelial cells leading to an upregulation in P-selectin expression and consequently elevating BBB permeability (49, 50).

The excessive increase in inflammatory factors following brain injury or disorder is the primary cause of BBB disruption (40). MCs represent a major source of these inflammatory factors, contributing to the disruption of the BBB in pathological conditions. For instance, the secretion of TNF-α by MCs can interact with the TNF-1 receptor or activate the NF-κB signaling pathway, thereby reducing the expression level of Claudin 5, leading to TJ disruption and subsequent BMEC death (51). Furthermore, IL-1β secreted by MCs disrupts the BBB through two mechanisms: firstly, it induces the expression of hypoxia-inducible factor-1 (HIF-1) and its gene target vascular endothelial growth factor-A (VEGF-A) in astrocytes, thus initiating BBB disruption (52). Secondly, IL-1β promotes the secretion of IL-6 and TNF-α, disrupting the paracellular pathway of BBB cells and increasing cell paracellular permeability (53).

## 5 Mast cells and brain disorders

### 5.1 Ischemic stroke

**Figure 3:**
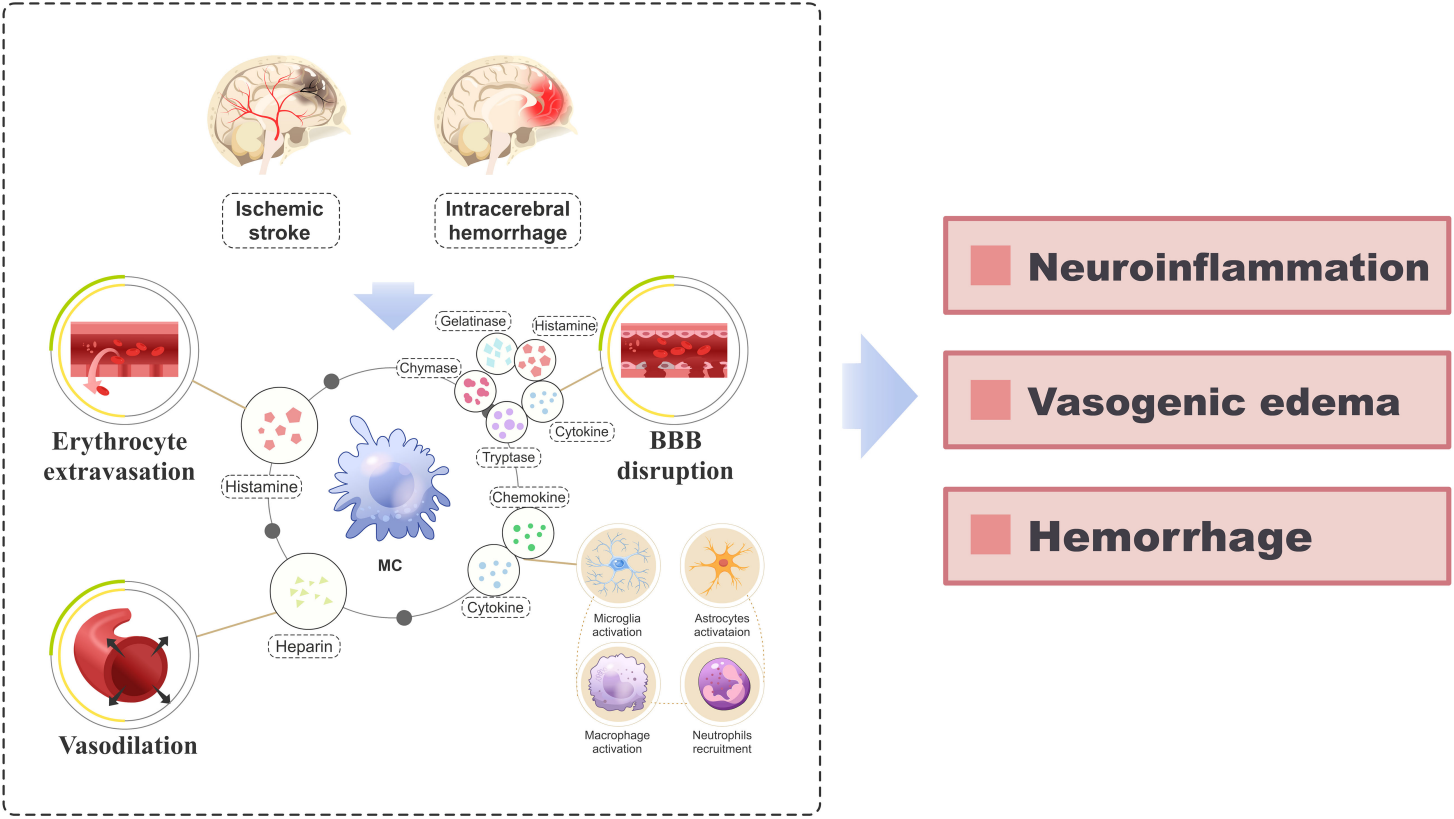
A schematic diagram elucidating the role of MCs in the pathogenesis of IS and ICH. After the occurrence of IS and ICH, activated MCs release a diverse array of vasoactive substances, cytokines, and proteolytic enzymes (including histamine, heparin, IL-6, TNF-α, IL-1β, tryptase, chymase, MMPs), which orchestrate erythrocyte extravasation, vasodilation and disruption of the BBB. Additionally, the release of various cytokines and chemokines from MCs triggers the recruitment and activation of neural immune cells (including microglia, astrocytes, macrophages, and neutrophils) from the periphery, perpetuating the inflammatory response. This pathological process ultimately culminates in the development of neuroinflammation, vasogenic edema, and hemorrhage. BBB, blood-brain barrier; IS, ischemic stroke; ICH, intracerebral hemorrhage; IL, interleukin; MC, mast cell; MMP, matrix metalloproteinases; TNF-α, tumor necrosis factor-alpha.

Ischemic stroke (IS) is the predominant form of stroke, accounting for approximately 87% of all stroke cases (54). The injuries associated with IS primarily encompass BBB disruption (55), oxidative stress (56), excitotoxicity (57), microvascular impairment (58), and neuroinflammation (59–62), ultimately leading to neuronal apoptosis (63). MCs play a crucial role in the pathogenic mechanisms of IS (**Figure 3**). Following IS, MCs perceive danger signals from the ischemic brain as early as 2-4 hours before microglia and astrocytes are activated (12, 64). They are among the first responders to be activated, contributing to disease progression and exacerbating neural damage by facilitating blood-brain barrier disruption and inflammatory infiltration (65). Relevant studies have demonstrated that rats with IS treated with MC activators exhibited a significant increase in brain edema, whereas those treated with MC stabilizers or MC-deficient rats showed reduced brain edema (65). Mast cells secrete TNF-α and tissue-type plasminogen activator, which can respectively augment the secretion of gelatinase by neighboring cells (66, 67) and activate MMP-9, thereby facilitating the disruption of the BBB (66, 68), hastening the progression of cerebral edema and increasing the risk of IS rat mortality (16). Furthermore, neutrophil infiltration commences several hours post Ischemia/Reperfusion (I/R) (69, 70), further exacerbating the accumulation of MMP-9 in microvessels (71), a process intricately linked to MCs (65). There are also reports in the literature indicating that following IS, meningeal MCs secrete IL-6, which can significantly exacerbate cerebral edema, enlarge the infarction area, and increase the number of granulocytes and activated macrophages in the brain (72). This finding is consistent with early clinical studies, where an elevated level of IL-6 in the cerebrospinal fluid of stroke patients was positively correlated with the size of the infarction area (73). Moreover, considering MCs’ strategic positioning near important blood vessels around the meninges and their interaction with cerebral circulation, they may function as potential “gatekeepers” to regulate immune cell infiltration into the brain during a stroke event (72).

Recently conducted studies have provided novel insights into the targeted treatment of MCs in IS. Previous research has demonstrated the anti-inflammatory and neuroprotective properties of N-Palmitoylethanolamide-Oxazoline (PEA-OXA) (74, 75). Fusco et al. demonstrated that PEA-OXA significantly attenuates the activation and detachment of MCs, as well as the NF-κB pathway activation in IS rats, leading to a reduction in I/R-related lesion size, cytokine expression, and histological damage (76). In recent years, researchers have increasingly focused on the significant role of the brain-gut axis in stroke (77–79). Following IS, there is a rise in the number of MCs in the intestines of mice, accompanied by elevated levels of histamine and pro-inflammatory cytokines in the brain and circulating plasma (79), which are closely associated with neurological prognosis (80). Inhibiting MCs during IS can alleviate cerebral and intestinal MCs activation, neuroinflammation, peripheral inflammation, reduce neurological deficits, and improve prognosis (81). Furthermore, recent studies have identified high expression of the PTGS2 gene, linked to MCs activation specifically in IS. Inhibiting PTGS2 has been shown to decrease infarction volume and neurological deficits while improving neurological prognosis in IS rats. This indirectly confirms that MCs may play a pivotal role in IS pathogenesis, suggesting that targeting genes related to MCs activation could lead to new breakthroughs in IS treatment (82).

### 5.2 Intracerebral hemorrhage

Intracerebral hemorrhage (ICH) refers to non-traumatic bleeding occurring in the brain parenchyma (83). Brain injury following ICH encompasses primary injury caused by hematoma compression and stimulation, as well as secondary brain injury such as blood-brain barrier disruption, neuroinflammation, oxidative stress, cell autophagy and apoptosis (84–86), among which the severity of secondary brain injury is closely linked to prognosis (87). The role of MCs in the early stage of secondary brain injury after ICH and throughout the course of the disease has been demonstrated by numerous studies (88) (**Figure 3**). After ICH, the activation of MCs results in BBB damage, exacerbation of cerebral edema, worsening of neurological deficits, and increased mortality. However, inhibiting MC activation can alleviate these effects (89, 90). Furthermore, MCs release various immune and inflammatory mediators that directly contribute to neuroinflammation following ICH (88). It is worth noting that MCs also play a role in ICH rebleeding and hematoma expansion. Studies have demonstrated that MCs release endogenous anticoagulant heparin, which impairs coagulation function and leads to red cell extravasation, thereby contributing to hematoma expansion (91, 92). Stabilizing or inducing deficiency in MCs can reduce the size of hematomas in ICH rats (89).

Several studies have investigated the inhibition of MCs activation following ICH. Inhalation of hydrogen has been shown to exert neuroprotective effects in ICH by reducing Lyn kinase phosphorylation, thereby inhibiting MCs activation and degranulation (93). Intravenous immunoglobulin (IVIG) can inhibit MCs activation after ICH by activating the FcγRIIB/SHIP1/PIP3 pathway, thus reducing neuroinflammation and improving BBB permeability (94). Yang et al.’s study demonstrated that inhibition of the IRE1α/miR-125/Lyn signaling pathway can reduce MCs activation, degranulation, and neuroinflammation after ICH, leading to reduced cerebral edema, hematoma size, and improved neurological deficits (95). Recent studies have demonstrated that GW0742 can attenuate neuroinflammation induced by MCs in rats with GMH (Germinal matrix hemorrhage) through activation of the PPARβ/δ/CD300a/SHP1 pathway (96). In conclusion, MCs play a role in the complex pathological process of ICH, and targeting MCs may offer new prospects for ICH treatment. Nevertheless, the pathophysiological significance of MCs in ICH remains incompletely understood, necessitating further investigation.

### 5.3 Intracranial aneurysm and subarachnoid hemorrhage

Intracranial aneurysm (IA) is a complex condition characterized by pathological dilation of cerebral arteries (97, 98). Subarachnoid hemorrhage (SAH) represents a severe consequence of IA rupture, associated with high mortality and morbidity rates (99, 100). Ample evidence suggests that MCs play a significant role in the pathophysiology of both IA and SAH.

MCs contribute to the IA through diverse mechanisms (101, 102). The intact IA vessel wall harbors a substantial population of activated MCs, which orchestrate vascular inflammation by upregulating the expression of inflammation-related molecules (103–105). Additionally, MCs secrete tryptase and chymase, leading to the upregulation of MMP-2 and MMP-9 expression and activity, thereby contributing to the degeneration of the arterial wall extracellular matrix (106–108). This cascade further induces the expression of inducible nitric oxide synthase (iNOS), which in turn mediates smooth muscle cell apoptosis. Consequently, this process results in decreased arterial stiffness, medial thinning, and ultimately culminates in IA expansion (105). Moreover, MCs can induce the formation and remodeling of new blood vessels in IA by secreting a variety of angiogenic factors (109, 110). Additionally, the histamine and proteases released by MCs can respectively facilitate leakage (111) and rupture of newly formed blood vessels (109, 110, 112), ultimately resulting in microbleeding.

Analysis of human IAs revealed a significantly higher degree of MC infiltration in ruptured IAs compared to unruptured IAs (103). Activation of MCs markedly increased the rate of IA rupture, while stabilizing MCs or correcting MC defects significantly reduced the incidence of IA rupture in mice (113). Stromal Cell-Derived Factor-1 (SDF-1), a well-known chemokine for MCs, can induce pathological remodeling of the IA wall (114). Meanwhile, the MCs in the IA wall secrete tryptase, which can convert angiotensin I into angiotensin II, thus activating the renin-angiotensin system and promoting the rupture of the IA (115, 116). In addition, the TNF-α and Hepatocyte Growth Factor (HGF) released by MCs also play a crucial role in the rupture of the IA (117, 118).

IA rupture is highly prone to causing SAH. Inflammation plays a crucial role in the pathogenesis of SAH (119) and is also a pivotal determinant of prognosis (120). Following SAH, MCs not only directly contribute to inflammatory damage by releasing various inflammatory mediators but also activate microglia to exert a synergistic pro-inflammatory effect (47). Cerebral vasospasm is a common complication following SAH and significantly increases the risk of delayed ischemia, leading to poor prognosis (121). The adenosine A3 receptor (A3R), abundantly expressed on the membranes of MCs, is responsible for vascular constriction (122). Meanwhile, the activation of A3R can also exacerbate brain injury by inducing the release of tryptase, chymase, pro-inflammatory cytokines, and chemokines from MCs, resulting in the infiltration of inflammatory cells into the injured hemisphere (123).

In recent years, numerous scholars have conducted research on MC as a therapeutic target. Studies have revealed that intravenous injection of MSCs can activate the cyclooxygenase-2 (COX-2)-dependent pathway, suppress MC activation, and effectively prevent IA rupture (124). Simultaneously, the microvesicles from intravenous injection of MSCs can also enhance the levels of prostaglandin E2 (PGE2) and E-prostanoid 4 (EP4) receptor expression associated with MCs in the IA, thereby safeguarding the vascular wall and preventing IA rupture (125). After SAH, the inhibition of MCs has been shown to significantly reduce cerebral edema, neuroinflammation, and neurological deficits (47). Moreover, LJ529 has demonstrated the ability to inhibit MC degranulation through the A3R-PKCϵ-ALDH2 pathway, thereby mitigating MC-related inflammation in SAH (126). In addition to these preclinical studies, it is well-established that various cytokines and chemokines are implicated in the pathogenic mechanism of MCs in IA and SAH; however, the specific mechanism remains incompletely elucidated. Therefore, further research on MCs is necessary to explore novel therapeutic approaches for IA and SAH treatment.

### 5.4 Alzheimer’s disease

Alzheimer’s disease (AD) is a neurodegenerative condition characterized by the prominent pathological features of extracellular amyloid-beta (Aβ) protein deposition, hyperphosphorylation of Tau protein, and synaptic loss leading to the development of neurofibrillary tangles (NFTs) and eventual neuronal death (127, 128). Research has demonstrated extensive MCs infiltration in the brains of AD patients, particularly in regions where Aβ protein accumulates (9, 129). This phenomenon is attributed to neuroglia cells within Aβ deposition sites releasing substantial amounts of chemoattractants for MC infiltration such as serum amyloid A. Furthermore, MCs themselves serve as early detectors of Aβ accumulation; Harcha et al. observed a rapid increase in MCs presence in the hippocampus and cortex prior to noticeable Aβ deposition (130).

**Figure 4:**
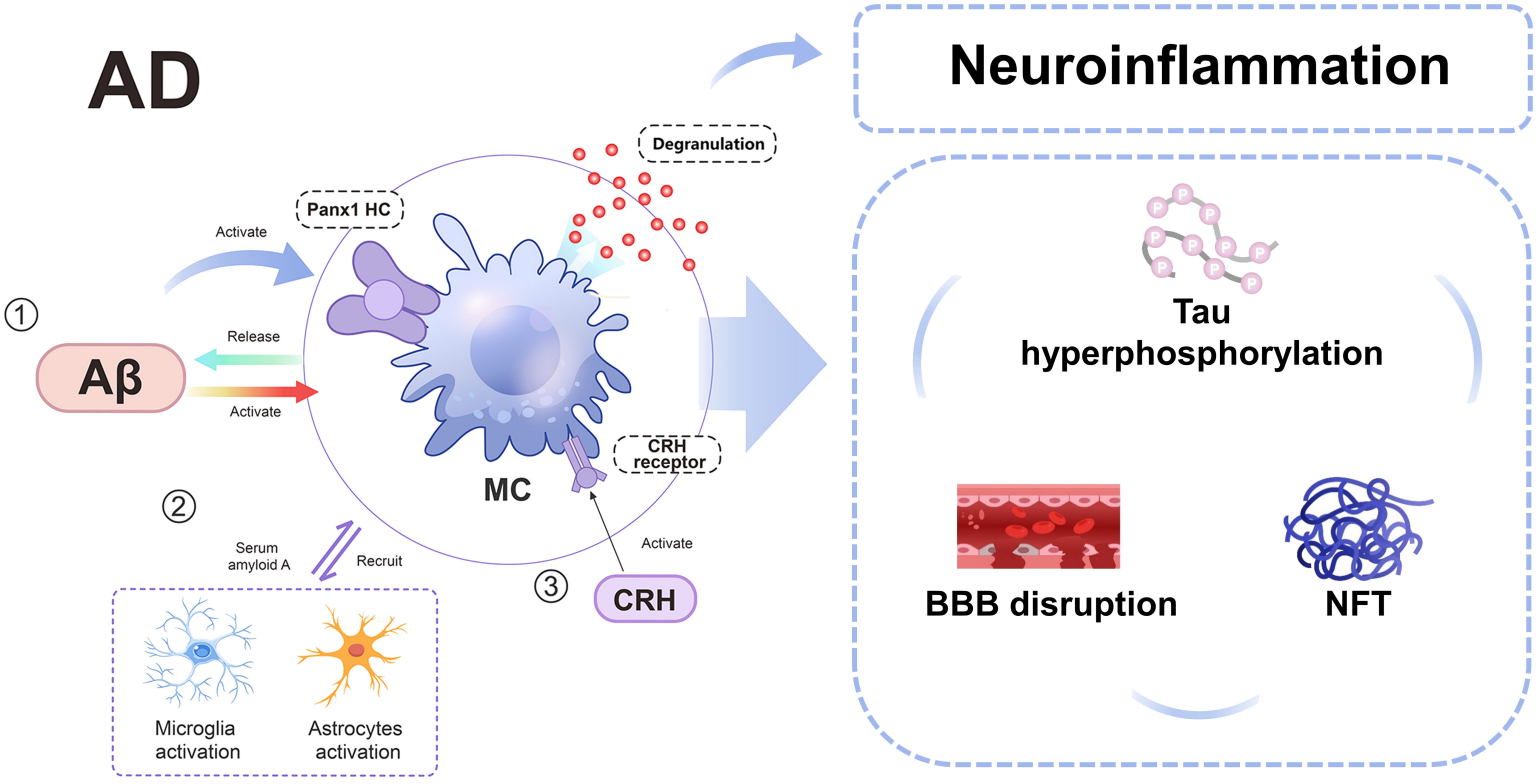
A schematic diagram illustrating the involvement of MCs in AD. In AD, MCs can be activated by Aβ, Panx1 HC, and CRH receptor, subsequently interacting with microglia and astrocytes. This interaction leads to disruption of the BBB, hyperphosphorylation of tau protein, and neurofibrillary tangles. AD, alzheimer’s disease; Aβ: amyloid-beta; BBB, blood-brain barrier; CRH, Corticotropin-releasing hormone; MC, mast cell; MMP, matrix metalloproteinases; NFT, neurofibrillary tangles; Panx1 HC, Pannexin1 hemichannel.

MCs have been demonstrated to contribute to the development of AD by promoting Aβ formation and neuroinflammation (131) (**Figure 4**). Activation of corticotropin-releasing hormone (CRH) receptors on MCs by CRH, through the hypothalamic-pituitary-adrenal axis, stimulates MC activation and disrupts the BBB, facilitating the entry of peripheral inflammatory factors and cells into the brain, thereby activating neuroglia cells (132). This process also leads to Aβ formation and subsequent tau protein aggregation, NFT formation, ultimately contributing to AD development (133). Additionally, deposited Aβ can induce degranulation by activating Pannexin1 hemichannel (Panx1 HC) on MCs and recruit other immune cells to release substantial amounts of inflammatory factors, thus participating in AD-related neuroinflammation (130). Recent studies have revealed that various forms of Aβ including Aβ1-42, Aβ1-40, and Aβ-35 can stimulate MCs to secrete significant levels of pro-inflammatory cytokines; among these forms, Aβ1-42 exhibits the most potent effect (134).

Recently, scholars have investigated the potential of MCs as a therapeutic target for AD. Lin et al. demonstrated that MC depletion in 5XFAD mice upregulated transcriptomic features of neuroprotective DAM, downregulated markers of reactive astrocytes, and improved hippocampal-dependent cognitive function (135). Furthermore, they discovered that dura mater MCs can survey cerebrospinal fluid to receive signals from the intracranial environment and respond by expressing immunomodulatory mediators that impact cognitive and neuroglial function (135). The tyrosine kinase inhibitor MC stabilizer masitinib has been demonstrated to exert neuroprotective effects by inhibiting the upregulation of BBB permeability by MCs and suppressing neuroinflammation at the onset of AD (136). These findings offer a preliminary foundation for exploring novel therapeutic targets for AD, but further research is necessary to elucidate and validate them.

### 5.5 Parkinson’s disease

Parkinson’s disease (PD) is an irreversible neurodegenerative condition characterized by the degeneration and loss of dopamine neurons in the substantia nigra pars compacta of the midbrain, along with the accumulation of misfolded α-synuclein (α-Syn), which forms eosinophilic inclusion bodies known as Lewy bodies (LB). These pathological changes are accompanied by neuroinflammation (137, 138).

**Figure 5:**
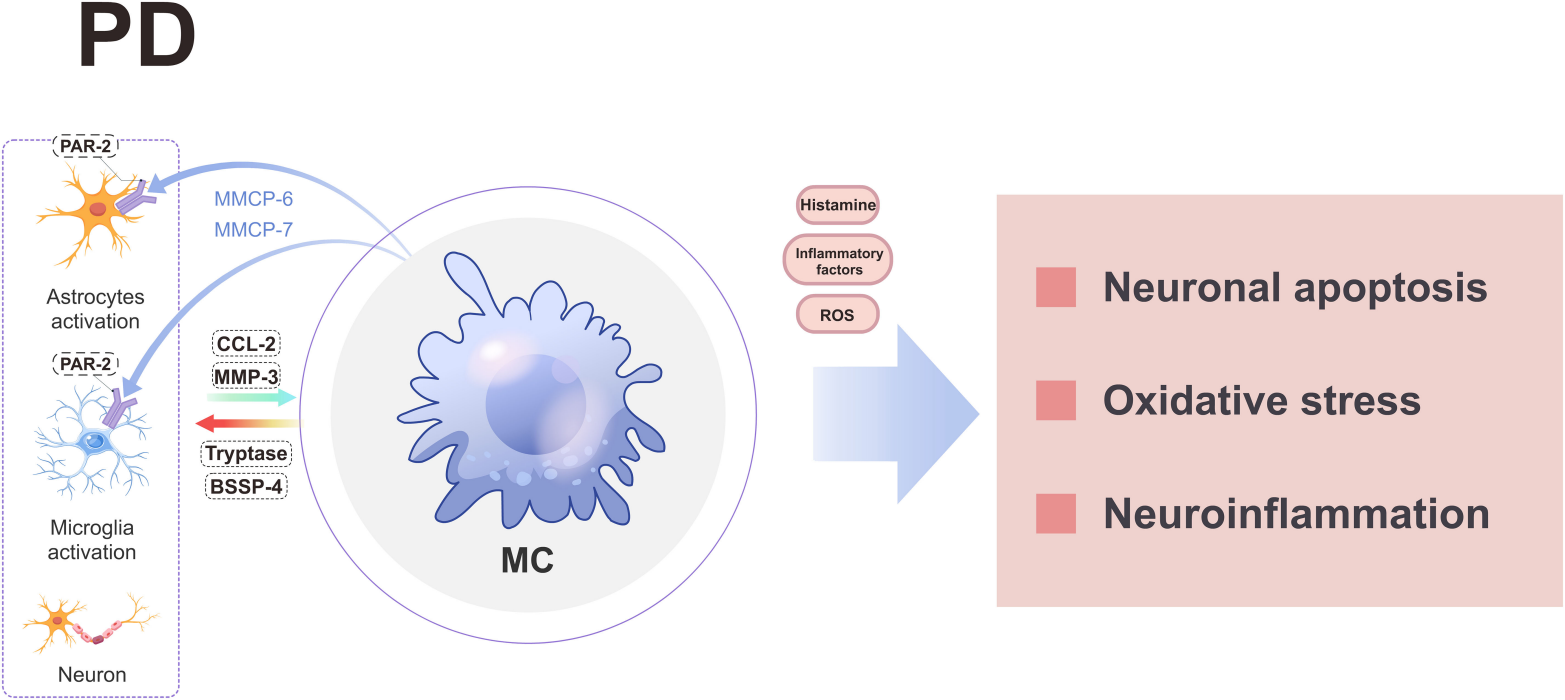
A schematic diagram illustrating the involvement of MCs in PD. MCs engage in interactions with neuroglial cells and neurons, leading to the release of various substances that ultimately induce neuroinflammation, oxidative stress, and neuronal apoptosis through the secretion of histamine, inflammatory factors, and ROS. BSSP-4, Brain-Specific Serine Protease-4; CCL-2, chemokine ligand 2; MC, mast cell; PD, parkinson’s disease; PAR-2, protease activated receptor 2; ROS, reactive oxygen species.

MCs play a pivotal role in the pathogenesis of PD through diverse mechanisms (**Figure 5**). In the context of PD, activated MCs are capable of concomitantly releasing other pro-inflammatory mediators and substantial amounts of reactive oxygen species (ROS), thereby eliciting oxidative stress (6, 139, 140). This oxidative stress represents a fundamental contributor to neurodegeneration in PD (141). Histamine significantly contributes to PD pathogenesis. Elevated levels along with increased presence within substantia nigra have been observed both in PD patients as well as animal models (142–145). Local administration or systemic delivery has been shown to result in dopaminergic neuron death alongside worsening dyskinesia symptoms (21). The induction mechanism involves activation through H2R and H4R receptors leading to dopaminergic degeneration (146), while inhibiting H2R activation suppresses JNK/P38 phosphorylation thus reducing CASP3 activity which mitigates cellular apoptosis (146). Furthermore, blocking H4R hinders MCs activation within brain tissue resulting in reduced TNF-α release thereby ameliorating neurodegeneration including LB-like neuropathological changes (147, 148). Notably, MCs serve as a primary source accounting for over 50% total brain-histamine secretion (14), suggesting potential therapeutic promise lies within regulating their secretory function for future PD treatments.

MCs may also contribute to the pathogenesis of PD by interacting with neuralglia cells and neurons. When MCs are co-cultured with neuralglia cells/neurons and exposed to glia activating factors, activated MCs can secrete tryptase and brain-specific serine protease-4 (BSSP-4) to stimulate the release of CC2L and MMP-3 by astrocytes and neurons, thereby contributing to the pathogenesis of PD (149). Meanwhile, MCs can enhance the expression of PAR-2 in neuroglia cells and neurons by releasing specific proteases (such as MMCP-6 and MMCP-7), leading to neuroinflammation and neuronal apoptosis (32). Conversely, neuroglia cells-secreted CCL2 can attract MCs to the substantia nigra region, triggering the release of diverse pro-inflammatory mediators and subsequent dopaminergic neuron death (150). Therefore, targeting MCs holds promise for novel therapeutic strategies in PD.

### 5.6 Amyotrophic lateral sclerosis

Amyotrophic lateral sclerosis (ALS) is a neurodegenerative disorder characterized by the degeneration of motor neurons and dysfunction of distal motor axons (151–153). Early studies have observed the activation of MCs in the degenerative regions of the central nervous system, such as the prefrontal cortex and pyramidal tract, as well as in the spinal cord of ALS patients (154, 155). Furthermore, Trials et al. discovered activated MCs in the skeletal muscles of ALS rats, which were found in the motor axons of the extensor digitorum longus muscle, sciatic nerve, and ventral root of the spinal cord (156, 157). These findings suggest that MCs play a role in ALS development by potentially inducing motor axon degeneration and accelerating neuromuscular junctions (NMJs) loss, thereby contributing to peripheral motor pathway degeneration in ALS patients (156, 157).

Studies indicate that MCs may also contribute to the vascular-related pathological mechanisms of ALS (158, 159). MCs play a crucial role in enhancing vascular permeability through the secretion of tryptase and chymase, which degrade adhesion protein complexes between endothelial cells (160), as well as connexin, procollagen, and type IV collagen in the extracellular matrix (161, 162). Furthermore, MCs release histamine and prostaglandin, further augmenting microvascular permeability (163), thereby facilitating the infiltration of peripheral inflammatory cells. These processes result in damage to microvascular endothelial cells and perivascular cells in the brains and spinal cords of ALS patients, leading to impairment of the BBB and blood-spinal cord barrier (164–166). Subsequently, MCs can breach the blood-spinal cord barrier (167, 168) and release neuropeptides, proteases, cytokines, and histamine, inducing local neuroinflammation and neuronal dysfunction (9).

MCs may also facilitate disease progression through interactions with other immune cells and neurons. They secrete chymase that promote neutrophil infiltration, contributing to the advancement of ALS paralysis (157). Furthermore, MCs can engage with microglia by secreting IL-6 and tryptase, establishing a positive feedback loop that continuously amplifies ALS neuroinflammation (9). Additionally, reactive astrocytes in ALS may express stem cell factor (SCF), which induces c-kit+ precursor MC differentiation and drives their migration from the periphery through the microvasculature to the spinal cord, where they localize and exacerbate the disorder (159). Under the influence of 75-kD neurotrophin receptors (p75NTR) abnormally expressed by damaged motor neurons, MCs can also induce apoptotic signaling (169). Therefore, these interactions between MCs and immune cells as well as neurons represent an important link in the pathogenesis of ALS.

Research has demonstrated that the MC inhibitor matsitini can decrease the population of MCs in the extensor digitorum longus muscle, thereby reducing the occurrence of NMJ denervation and motor deficits in ALS rats, as well as ameliorating ALS neurological symptoms (156, 157). This substantiates the specific involvement of MCs in ALS, and the positive outcomes from a phase III clinical trial of matsitini in ALS further support its therapeutic potential, indicating promising prospects for targeting MCs as a treatment strategy for ALS (170). Nevertheless, there is still a need for comprehensive research to delve into the specific mechanisms through which MCs are implicated in ALS.

## 6 Conclusion and future perspectives

In recent years, with the continuous deepening of research, we have gained a more profound comprehension of the neuroprotective function of MCs and its significance in various brain disorders. On one hand, MCs can induce neuroinflammation and oxidative stress, promote BBB damage, vascular edema, and hemorrhage formation, as well as recruit other immune cells to exacerbate inflammatory responses. On the other hand, the potentially beneficial effects of activated MCs on the brain cannot be disregarded. MCs can exert neuroprotective effects by secreting histamine, NGF, and angiogenic factors. Similar to microglia and macrophages, brain MCs may contribute to neuroprotection and repair in the advanced stages of brain disorders.

It is important to acknowledge that numerous obstacles must be overcome in order to propel research in this domain. First, the activation of MCs in the brain is a complex process that may give rise to both neuroprotective and pathogenic effects. Our current understanding of the transcriptional and epigenetic dynamics governing MC activation in the brain is still in its early stages, necessitating further exploration into the regulation of MC activation. Second, previous studies have predominantly focused on directly inhibiting MC activation in brain disorder models without delving into specific underlying mechanisms. Therefore, gaining a deeper comprehension of the cell-to-cell interactions between MCs and other immune cells in the brain is essential for effectively modulating the brain’s immune response network. Third, while targeted interventions for MCs have demonstrated promising therapeutic potential in most brain disorders during preclinical studies, there remains controversy surrounding the role, mechanism, and potential therapeutic value of MCs in certain brain disorders. This highlights an urgent need to investigate the precise functional roles of MCs within brain pathology. Finally, Intestinal MCs can engage with neurons and endocrine elements via the brain-gut axis, thereby modulating immune responses and neuroinflammation linked to neurodegenerative conditions in the brain. From this perspective, targeting MCs as a therapeutic approach holds significant promise; employing strategies to inhibit their activity may offer a novel perspective for treating brain disorders as disorder-modifying therapy.

In conclusion, MCs are progressively emerging as a novel target for the treatment of brain disorders. A comprehensive exploration of the underlying molecular mechanisms through mechanistic research and identification of potential therapeutic interventions is poised to catalyze breakthroughs in the management of brain disorders.
